# α_1A_-adrenaline receptors in dorsal horn inhibitory neurons have an inhibitory role in the regulation of chloroquine-induced itch in mice

**DOI:** 10.1186/s13041-021-00768-9

**Published:** 2021-03-16

**Authors:** Yuto Shiraishi, Keisuke Koga, Ryo Yamagata, Izuho Hatada, Miho Shiratori-Hayashi, Makoto Tsuda

**Affiliations:** 1 Department of Life Innovation, Graduate School of Pharmaceutical Sciences, Kyushu University, Fukuoka, 812-8582 Japan; 2 Department of Neurophysiology, Hyogo College of Medicine, Nishinomiya, Hyogo 663-8501 Japan; 3 Department of Molecular and System Pharmacology, Graduate School of Pharmaceutical Sciences, Kyushu University, 3-1-1 Maidashi, Higashi-ku, Fukuoka, 812-8582 Japan; 4 Laboratory of Genome Science, Biosignal Genome Resource Center, Institute for Molecular and Cellular Regulation, Gunma University, 3-39-15 Showa-machi, Maebashi, Gunma 371-8512 Japan

**Keywords:** α_1A_-Adrenaline receptor, Spinal dorsal horn, Inhibitory interneurons, Itch, Mouse

## Abstract

Our previous study showed the intrinsic ability of descending noradrenergic neurons projecting from the locus coeruleus to the spinal dorsal horn (SDH) to suppress itch-related behaviors. Noradrenaline and α_1A_-adrenaline receptor (α_1A_-AR) agonist increase inhibitory synaptic inputs onto SDH interneurons expressing gastrin-releasing peptide receptors, which are essential for itch transmission. However, the contribution of α_1A_-ARs expressed in SDH inhibitory interneurons to itch-related behavior remains to be determined. In this study, RNAscope in situ hybridization revealed that *Adra1a* mRNA is expressed in SDH inhibitory interneurons that are positive for *Slc32a1* mRNA (known as vesicular GABA transporter). Mice with conditional knock-out of α_1A_-ARs in inhibitory interneurons (*Vgat-Cre*;*Adra1a*^flox/flox^ mice) exhibited an increase in scratching behavior when induced by an intradermal injection of chloroquine, but not compound 48/80, which are known as models of histamine-independent and dependent itch, respectively. Furthermore, knockout of inhibitory neuronal α_1A_-ARs in the SDH using the CRISPR–Cas9 system also increased the scratching behavior elicited by chloroquine but not compound 48/80. Our findings demonstrated for the first time that α_1A_-ARs in SDH inhibitory interneurons contribute to the regulation of itch signaling with preference for histamine-independent itch.

**Supplementary Information:**

The online version contains supplementary material available at 10.1186/s13041-021-00768-9.

Itch is defined as an unpleasant sensation that provokes a desire to scratch to transiently attenuate such sensations. Sensory itch information from the cutaneous nerve endings of primary afferent fibers is conveyed to the spinal dorsal horn (SDH). Recent studies have made great progress in furthering our understanding of the neuronal circuits for itch transmission in the SDH [1]. Specifically, gastrin-releasing peptide receptor (GRPR)-expressing (GRPR^+^) neurons in the SDH have been identified as an essential component of spinal itch transmission [2]. Furthermore, it has also been shown that GRPR^+^ neurons are controlled not only locally by subsets of excitatory and inhibitory interneurons in the SDH [3] but also remotely by descending monoaminergic neurons from the brainstem [4, 5]. A recent study has shown that the activation of descending serotonergic (5-HTergic) neurons facilitates GRPR signaling and itch transmission in the SDH via activation of 5-HT_1A_ receptors [4]. As for noradrenaline (NA), we recently demonstrated that NAergic neurons descending from the locus coeruleus (LC) to the SDH negatively control itch-related scratching behaviors. Furthermore, application of either NA or an α_1A_-adrenaline receptor (α_1A_-AR) agonist to spinal cord slices facilitates the transmission of inhibitory synaptic inputs into GRPR^+^ SDH neurons [5]. Thus, it is possible that α_1A_-ARs in inhibitory SDH interneurons may play a role in controlling itch-related behavior, although this is entirely unknown. The aim of this study was to determine the role of α_1A_-ARs expressed in inhibitory SDH interneurons using genetic tools that enable cell- and region-specific gene manipulations.

We generated mice in which α_1A_-ARs are conditionally knocked out in inhibitory neurons by crossing *Vgat-Cre* mice (in which Cre is expressed in inhibitory neurons under the control of the promoter of vesicular GABA transporter [6]) with the *Adra1a*^flox/flox^ mice that we recently developed [7] (Additional file 1). Scratching behavior was induced by injecting pruritogens (compound 48/80 and chloroquine, models of histamine-dependent and -independent itch, respectively [2, 8]) intradermally into the nape of the neck of *Adra1a*^flox/flox^ and *Vgat-Cre*;*Adra1a*^flox/flox^ mice. The scratching responses induced by compound 48/80 were indistinguishable between *Adra1a*^flox/flox^ and *Vgat-Cre*;*Adra1a*^flox/flox^ mice; however, the chloroquine-induced scratching responses were significantly enhanced in *Vgat-Cre*;*Adra1a*^flox/flox^ mice compared to *Adra1a*^flox/flox^ mice (Fig. 1a). In addition, nociceptive behavior (wiping responses to the capsaicin-injected cheek) was not different between the two genotypes (*Adra1a*^flox/flox^ mice, 46.0 ± 9.6, n = 5; *Vgat-Cre*;*Adra1a*^flox/flox^ mice, 40.2 ± 9.7, n = 6; P = 0.623, unpaired t-test). Our previous study showed that the application of α_1A_-AR agonist to spinal cord slices facilitates the transmission of inhibitory synaptic inputs into GRPR^+^ SDH neurons, raising the possibility that α_1A_-ARs in inhibitory SDH interneurons have a role in the itch response [5]. We examined the expression of α_1A_-ARs in the SDH using RNAscope in situ hybridization and found that *Adra1a* mRNA was clearly detected in inhibitory interneurons positive for *Slc32a1* mRNA (also known as *Vgat*) in the SDH of control *Adra1a*^flox/flox^ mice (Fig. 1b, c). Quantitative analysis revealed that 18.3% of *Slc32a1*^+^ SDH interneurons were positive for *Adra1a* mRNA (Fig. 1c). SDH cells expressing both *Adra1a* and *Slc32a1* mRNA were almost absent in *Vgat-Cre*;*Adra1a*^flox/flox^ mice (Fig. 1b, c), confirming the specificity of the probe to detect *Adra1a* mRNA and suggesting that α_1A_-ARs are expressed in inhibitory SDH interneurons. Because *Vgat-Cre*;*Adra1a*^flox/flox^ mice might lack α_1A_-AR expression in inhibitory neurons in the brain, we employed the CRISPR–Cas9 system using adeno-associated virus (AAV) vectors [9] to knock out inhibitory neuronal α_1A_-ARs specifically in the SDH. Cre-dependent *Staphylococcus aureus* Cas9 (SaCas9)-expressing vectors (AAV-FLEX-SaCas9) and single guide RNA-expression vectors (AAV-FLEX-mCherry-U6-sgAdra1a or -sgRosa [as control]) were injected into the bilateral cervical SDH of *Vgat-Cre* mice (Fig. 1d). *Vgat-Cre* mice injected with AAV-FLEX-SaCas9 and AAV-FLEX-mCherry-U6-sgAdra1a did not show changes in scratching behavior as elicited by compound 48/80; however, they showed a significant increase in scratching when itch was induced by chloroquine (Fig. 1e).

**Fig. 1:**
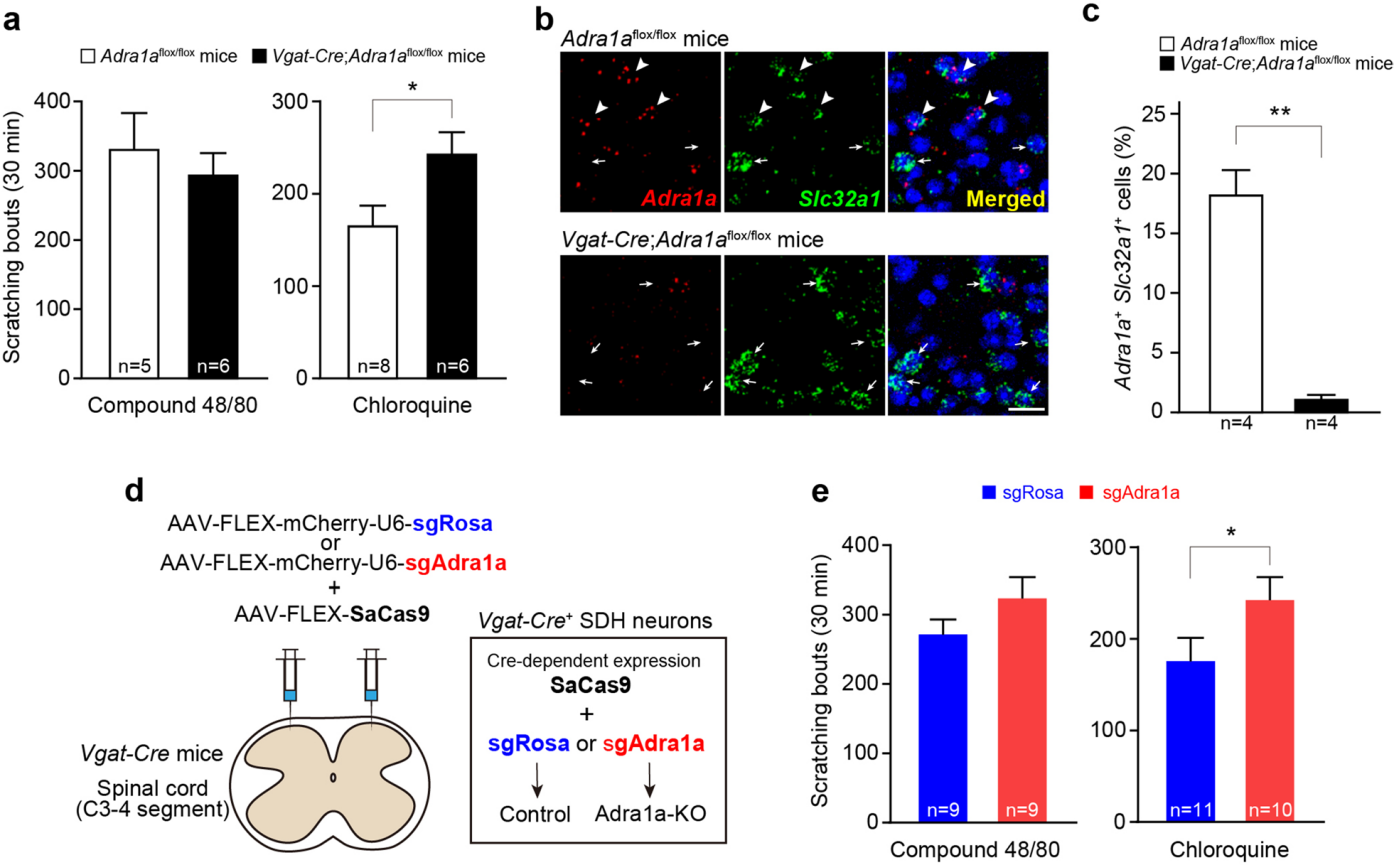
Knockout of α_1A_-ARs in inhibitory interneurons enhances scratching behavior evoked by chloroquine. **a** Scratching behaviors induced by intradermal injection of compound 48/80 (50 μg/50 μl) or chloroquine (200 μg/50 μl) in *Adra1a*^flox/flox^ mice or *Vgat-Cre*;*Adra1a*^flox/flox^ mice. *P < 0.05. **b** RNAscope in situ hybridization for mRNAs of *Adra1a* (red) and *Slc32a1* (green) in the SDH of *Adra1a*^flox/flox^ mice or *Vgat-Cre*;*Adra1a*^flox/flox^ mice. 4,6-diamidino-2-phenylindole (DAPI) to mark nuclei is blue. Arrowheads and arrows indicate *Adra1a*^+^ *Slc32a1*^+^ cells and *Adra1a*^–^ *Slc32a1*^+^ cells, respectively. Scale bar, 20 μm. **c** Percentage of *Adra1a*^+^ *Slc32a1*^+^ cells per total *Slc32a1*^+^ cells tested in the SDH of *Adra1a*^flox/flox^ mice or *Vgat-Cre*;*Adra1a*^flox/flox^ mice. **P < 0.005. **d** Schematic illustration of transduction strategy of SaCas9 and guide RNA (sgAdra1a and sgRosa (control)) expression in SDH inhibitory neurons. **e** Effect of genome editing on scratching behaviors induced by intradermal injection of compound 48/80 (50 μg/50 μl) or chloroquine (200 μg/50 μl). *P < 0.05. Data show the mean ± SEM

By generating two lines of genetically manipulated mice in a cell type- and region-specific manner, we demonstrate for the first time that α_1A_-ARs in SDH inhibitory interneurons play an inhibitory role in the regulation of itch-related behavioral responses. The present data reinforce and extend our previous findings indicating an intrinsic inhibitory control for spinal itch transmission by SDH-projecting LC-NAergic neurons [5]. An unexpected and interesting finding of the present study was that the knockout of α_1A_-ARs in SDH inhibitory interneurons enhanced scratching behavior elicited by chloroquine but not by compound 48/80, whereas silencing SDH-projecting LC-NAergic neurons is known to enhance scratching induced by both pruritogens [5]. Given that chloroquine and compound 48/80 cause histamine-independent and -dependent itch, respectively [2, 8], it is possible that α_1A_-ARs in SDH inhibitory interneurons preferentially contribute to the regulation of histamine-independent itch. This study showed that *Adra1a* was expressed in SDH inhibitory interneurons, and activation of spinal α_1A_-ARs has been shown to facilitate the transmission of inhibitory synaptic inputs onto GRPR^+^ SDH neurons [5]. It is thus conceivable that inhibitory signals acting on GRPR^+^ neurons via α_1A_-AR-expressing SDH inhibitory interneurons suppress histamine-independent itch (Additional file 2). Other ARs, such as α_2_-ARs in GRPR^+^ neurons [10] and at primary afferent terminals [11], may be involved in histamine-dependent itch. However, scratching behaviors evoked by chloroquine and compound 48/80 have been shown to be dramatically suppressed in mice with ablated GRPR^+^ SDH neurons [2]. Recent single-cell transcriptome analysis in SDH neurons revealed the presence of several putative subsets expressing *Grpr* mRNA [10]. Thus, it is speculated that histamine-independent itch transmission may be relayed through a subset of GRPR^+^ neurons that is regulated by α_1A_-AR-expressing SDH inhibitory interneurons (Additional file 2). Alternatively, considering previous findings that showed that GRPR-knockout mice exhibit clearer suppression of scratching behaviors evoked by chloroquine than by compound 48/80 [12], and that these mice fail to suppress histamine-dependent itch [2], interactions between signals from GRPRs and GABA (and glycine) receptors in GRPR^+^ SDH neurons may also occur.

Considering the data from single-cell transcriptome of SDH neurons, it appears that there are some subsets of *Adra1a*-expressing inhibitory interneurons including ones that express *Npy* mRNA [10]. However, SDH neurons expressing Cre under the control of the *Npy* promoter (NPY::Cre) have been reported to selectively contribute to mechanical (but not chemical) itch [13]. Furthermore, intrathecal administration of an Y1 receptor agonist has no effect on scratching caused by chloroquine [14]. Thus, it is hypothesized that a subset of α_1A_-AR-expressing inhibitory SDH interneurons that are negative to NPY expression could be involved in the regulation of histamine-independent itch transmission.

Although these are important points to be elucidated by further investigations, our findings reveal a selective role of α_1A_-ARs in SDH inhibitory interneurons in histamine-independent itch and provide a clue to understanding the neuronal circuits of spinal itch transmission.

## Supplementary Information

**Additional file 1:** Materials and methods.

**Additional file 2:** Schematic illustration of possible neuronal circuits regulated by α_1A_-ARs expressed in *Vgat-Cre*^+^ inhibitory interneurons in the SDH.

## Data Availability

All data generated or analyzed during this study are included in this published article and its Additional file.

## Abbreviations

SDH: Spinal dorsal horn
GRPR: Gastrin-releasing peptide receptor
GRPR^+^ neurons: Gastrin-releasing peptide receptor-expressing neurons
5-HTergic neurons: Serotonergic neurons
NA: Noradrenaline
LC: Locus coeruleus
α_1A_-AR: α_1A_ adrenaline receptor
AAV: Adeno-associated virus
SaCas9: *Staphylococcus aureus* Cas9
