# Supplementary material for: α1A-adrenaline receptors in dorsal horn inhibitory neurons have an inhibitory role in the regulation of chloroquine-induced itch in mice
Source: Mol Brain. 2021 Mar 16;14:55. doi: 10.1186/s13041-021-00768-9 (PMC7962300; doi:10.1186/s13041-021-00768-9)
Supplement: Supplementary file 1 — Additional file 1: Materials and methods. [file 13041_2021_768_MOESM1_ESM.docx]

**Additional file 1**

**Materials and methods**

**Animals**

Male *Vgat-Ires-Cre* knock-in mice (B6J.129S6 (FVB)-*Slc32a1^tm2(cre)lowl^*/MwarJ) [1], male and female *Adra1a*^flox/flox^ mice [2,3] were used. All mice used were 8–12 weeks of age at the start of each experiment and were housed at temperature and humidity ranges of 21–23 °C and 40–60%, respectively, with a 12-h light–dark cycle. All animals were fed food and water ad libitum. All animals were housed in standard polycarbonate cages in groups of same-sex littermates. All animal experiments were conducted according to relevant national and international guidelines contained in the Act on Welfare and Management of Animals (Ministry of Environment of Japan) and the Regulation of Laboratory Animals (Kyushu University) and under the protocols approved by the Institutional Animal Care and Use committee review panels at Kyushu University.

**RNAscope *in situ* hybridization**

RNAscope *in situ* hybridization was performed by the method described previously [3]. Mice were deeply anesthetized with an i.p. injection of pentobarbital and transcardially perfused with phosphate-buffered saline (PBS) followed by ice-cold 4% paraformaldehyde (PFA)/PBS. The C3–C4 segments of the spinal cord were removed, postfixed in the same fixative overnight at 4°C, and placed in 30% sucrose solution for 24–48 h at 4°C. Tissues were embedded in OCT compound (Sakura Finetek Japan) and sectioned at a thickness of 14 μm. Fluorescence *in situ* hybridization (ACDbio) was performed following the manufacturer’s instructions for fixed frozen tissue. The following probes were used: Mm-Slc32a1-C3 (ACDbio, 319191-C3), Mm-Adra1a (ACDbio, 408611). Tissue sections were analyzed using an LSM700 Imaging System (ZEN 2012, Carl Zeiss). Cells were considered positive if three or more punctate dots were present in the nucleus and/or cytoplasm [4].

**AAV vector construction and production.**

AAV vector plasmids of Cre-dependent gene transduction were generated from pAAV-Ef1a-DIO ChETA-EYFP (Addgene, #26968) [5], by substituting the ChETA-EYFP with mCherry or SaCas9. To reduce packaging size of Cre induced mCherry or SaCas9 expression vector, we generated pZac2.1-CMVmini-FLEX-mCherry-WPRE or pZac2.1-CMVmini-FLEX-SaCas9-WPRE from pZac2.1-CMVmini-SaCas9 vector (Addgene, #78601) [6], by substituting the SaCas9 with FLEX-SaCas9-WPRE or FLEX-mCherry-WPRE. Synthetic oligonucleotides including targeting sequence for exon2 of *Adra1a* (5’-ATGCCGATGACAGGCCACCGA-3’) with the targeting site in the original pENTER-U6-sgBsa1 plasmid [7]. This U6-sgRNA cassette and control guide targeting or for *Rosa* locus (5’-CTCTAGAGTCGCAGATCCTC-3’) [8] was transferred into pZac2.1-CMVmini-FLEX-mCherry-WPRE. The AAV vectors were produced from human embryonic kidney 293 (HEK293) cells with triple transfection (each pZac2.1 plasmid; pAAV2/9 trans plasmid; pAd DeltaF6, adenoviral helper plasmid). Viral lysate was harvested at 72 h post-transfection and lysed by freeze-and-thaw cycles, purified through two rounds of CsCl ultracentrifugation, and then concentrated using Vivaspin 20 ultrafiltration units (SARSTEDT, Germany). The genomic titer of rAAV was determined by Pico Green fluorometric reagent (Molecular Probes, USA) following denaturation of the AAV particle. Vectors were stored in aliquots at −80 °C until use.

**Intra-SDH and injection of rAAV vector**

Viral injection was performed as previously described [8,9]. Mice were deeply anesthetized by subcutaneous injection of ketamine (100 mg/kg) and xylazine (10 mg/kg). Mice was shaved on the back of the neck, and the skin was incised at C3–C5. The muscle on C3–C5 vertebrae was opened with a retractor, and mice were

attached with a head-holding device (SR-AR, NARISHIGE, Japan). Paraspinal muscles around the left side of the interspace between C3 and C4 vertebrae were removed, and the dura mater and the arachnoid membrane were carefully incised using the tip of a30G needle to make a small window to allow a glass microcapillary insert directly into the SDH. The glass microcapillary was inserted into the SDH (150–200 μm in depth from the surface of the dorsal root entry zone) through the small window (approximately 500 μm lateral from the midline). rAAV solution was pressure-ejected (100 nL/min) for 5 min (approximately 500 nL) using the Micro Syringe Pumps (SYSmicro4, WPI, USA) bilaterally. After microinjection, the inserted glass microcapillary was removed from the SDH, the skin was sutured with 3–0 silk, and mice were kept on a heating light until recovery. We used virus-injected mice for behavioral experiments 21 days or more after the last injection of AAV vectors. The used viral titers were as follows: AAV2/9-CMV-FLEX-SaCas9-WPRE, 1×10^12^ genome copies (GC)/ml; AAV2/9-CMV-FLEX-mCherry-U6-sgAdra1a-WPRE, 0.5×10^12^ GC/ml; AAV-2/9-CMV-FLEX-mCherry-U6-sgRosa-WPRE, 0.5×10^12^ GC/ml.

**Mouse models of acute itch**

Behavioral tests were performed by the methods in our previous study [8]. Mice were shaved on the rostral back until one day before injection of pruritogens. Intradermal injection of pruritogens [chloroquine (200 μg/50 μl; C6628, Sigma) and compound 48/80 (50 μg/50 μl; C2313, Sigma)] into the shaved rostral back was performed. After the injection, the mouse was placed in a plastic chamber (11 cm in diameter, 10 cm high). Hind limb scratching behavior directed toward the injection site was observed for 30 min. One scratch was defined as a lifting of the hind limb toward the injection site and then placing the limb back on the floor, regardless of how many scratching strokes took place between those two movements.

**Acute nociceptive behavior**

Mice were shaved on the right cheek until one day before experiments. Injection of capsaicin (10 µg/10 µl; M2028, Sigma) into the shaved cheek was performed. After the injection, the mouse was placed in a plastic chamber (11 cm in diameter, 10 cm high). Forelimb wiping behavior directed toward the injection site was observed for 20 min. One wiping was defined as single stroke that began at the back of the cheek, and moved forward in a caudal to rostral direction [10].

**Statistical analysis**

All data are shown as the mean ± SEM. Statistical significance of differences was

determined using unpaired t-test (Fig. 1a, 1e), unpaired t test with Welch's correction (Fig. 1c) using GraphPad Prism 4 software. Differences were considered significant at P<0.05.

**References**

1. Vong L, Ye C, Yang Z, Choi B, Chua S, Jr., Lowell BB. Leptin action on GABAergic neurons prevents obesity and reduces inhibitory tone to POMC neurons. Neuron 2011;71(1):142-154.

2. Horii T, Morita S, Kimura M, Terawaki N, Shibutani M, Hatada I. Efficient generation of conditional knockout mice via sequential introduction of lox sites. Sci Rep 2017;7(1):7891.

3. Kohro Y, Matsuda T, Yoshihara K, Kohno K, Koga K, Katsuragi R, et al. Spinal astrocytes in superficial laminae gate brainstem descending control of mechanosensory hypersensitivity. Nat Neurosci 2020;23(11):1376-1387.

4. Munanairi A, Liu XY, Barry DM, Yang Q, Yin JB, Jin H, et al. Non-canonical Opioid Signaling Inhibits Itch Transmission in the Spinal Cord of Mice. Cell Rep 2018;23(3):866-877.

5. Gunaydin LA, Yizhar O, Berndt A, Sohal VS, Deisseroth K, Hegemann P. Ultrafast optogenetic control. Nat Neurosci 2010;13(3):387-392.

6. Tabebordbar M, Zhu K, Cheng JKW, Chew WL, Widrick JJ, Yan WX, et al. In vivo gene editing in dystrophic mouse muscle and muscle stem cells. Science 2016;351(6271):407-411.

7. Ran FA, Cong L, Yan WX, Scott DA, Gootenberg JS, Kriz AJ, et al. In vivo genome editing using Staphylococcus aureus Cas9. Nature 2015;520(7546):186-191.

8. Koga K, Shiraishi Y, Yamagata R, Tozaki-Saitoh H, Shiratori-Hayashi M, Tsuda M. Intrinsic braking role of descending locus coeruleus noradrenergic neurons in acute and chronic itch in mice. Mol Brain 2020;13(1):144.

9. Koga K, Yamagata R, Kohno K, Yamane T, Shiratori-Hayashi M, Kohro Y, et al. Sensitization of spinal itch transmission neurons in a mouse model of chronic itch requires an astrocytic factor. J Allergy Clin Immunol 2020;145(1):183-191.e110.

10. Shimada SG, LaMotte RH. Behavioral differentiation between itch and pain in mouse. Pain 2008;139(3):681-687.
