# Supplementary material for: α1A-adrenaline receptors in dorsal horn inhibitory neurons have an inhibitory role in the regulation of chloroquine-induced itch in mice
Source: Mol Brain. 2021 Mar 16;14:55. doi: 10.1186/s13041-021-00768-9 (PMC7962300; doi:10.1186/s13041-021-00768-9)
Supplement: Supplementary file 2 — Additional file 2: Schematic illustration of possible neuronal circuits regulated by α1A-ARs expressed in Vgat-Cre+ inhibitory interneurons in the SDH. [file 13041_2021_768_MOESM2_ESM.docx]

**Additional file 2**

**Schematic illustration of possible neuronal circuits regulated by α_1A_-ARs expressed in *Vgat-Cre*^+^ inhibitory interneurons in the SDH**

Pruriceptive information from primary afferents is relayed through SDH interneurons including GRPR^+^ neurons. Based on our findings showing that (1) *Adra1a* was expressed in *Vgat-Cre*^+^ SDH inhibitory interneurons (Fig. 1b, c), (2) the knockout of α_1A_-ARs in these neurons enhanced scratching behavior elicited by chloroquine but not by compound 48/80 (Fig. 1a, e), and (3) activation of spinal α_1A_-ARs has been shown to facilitate the transmission of inhibitory synaptic inputs onto GRPR^+^ SDH neurons (Koga et al., Mol Brain 13: 144, 2020), it is hypothesized that inhibitory signals acting on GRPR^+^ neurons via α_1A_-AR-expressing SDH inhibitory interneurons suppress histamine-independent itch. On the other hand, because silencing SDH-projecting locus coeruleus-NAergic neurons has shown to enhance histamine-dependent and independent itch (Koga et al., Mol Brain 13: 144, 2020), other ARs such as α_2_-ARs in SDH neurons and at primary afferent terminals may also be involved in histamine-dependent itch.
